# Correlations between Iron Metabolism Parameters, Inflammatory Markers and Lipid Profile Indicators in Patients with Type 1 and Type 2 Diabetes Mellitus

**DOI:** 10.3390/jpm10030070

**Published:** 2020-07-25

**Authors:** Nadezhda N. Musina, Tatiana V. Saprina, Tatiana S. Prokhorenko, Alexander Kanev, Anastasia P. Zima

**Affiliations:** 1 Siberian State Medical University, 634050 Tomsk, Russia or tanja.v.saprina@mail.ru (T.V.S.); mmikld.ssmu@gmail.com (T.S.P.); alexkanev92@gmail.com (A.K.); zima2302@gmail.com (A.P.Z.); 2 Tomsk Regional Blood Center, 634034 Tomsk, Russia

**Keywords:** diabetes mellitus, anemia of chronic disease, iron deficiency anemia, hyperlipidemia, inflammation, C-reactive protein, tumor necrosis factor-α, erythrocyte sedimentation rate

## Abstract

This study aims to establish relationships between inflammatory status, ferrokinetics and lipid metabolism in patients with diabetes mellitus. Subclinical inflammation was assessed by levels of high-sensitive C-reactive protein, tumor necrosis factor-α and erythrocyte sedimentation rate. Iron metabolism parameters included complete blood count, serum iron, transferrin and ferritin. Metabolic status assessment included lipid profile, glycated hemoglobin and microalbuminuria measurement. As a result of the study it was possible to establish both general (universal) and diabetes mellitus (DM) type-dependent relationships between the parameters of lipid profile and metabolic control in DM. High-density lipoprotein cholesterol (HDL-C) levels negatively correlated with microalbuminuria (*r* = −0.293; *p* ˂ 0.05 for type 1 diabetes and *r* = −0.272; *p* ˂ 0.05 for type 2 diabetes). Ferritin concentration positively correlated with triglyceride level (*r* = 0.346; *p* ˂ 0.05 for type 1 diabetes and *r* = 0.244; *p* ˂ 0.05 for type 2 diabetes). In type 1 diabetes, a negative correlation was discovered between estimated glomerular filtration rate (eGFR) and LDL-C (*r* = −0.480; *p* ˂ 0.05), very low-density-lipoprotein cholesterol (VLDL-C) (*r* = −0.490; *p* ˂ 0.05) and triglycerides (*r* = −0.553; *p* ˂ 0.05), and a positive one between C-reactive protein concentration and triglyceride level (*r* = 0.567; *p* ˂ 0.05). Discovered relationships between lipid profile indices, inflammatory status and microalbuminuria confirmed mutual influence of hyperlipidemia, inflammation and nephropathy in diabetes patients. Obtained results justify the strategy of early hypolipidemic therapy in patients with diabetes mellitus to prevent the development and progression of microvascular complications.

## 1. Introduction

It is now well-established that diabetes mellitus (DM) is associated with a proinflammatory immune status and is accompanied by an increase in the level of circulating inflammatory markers. Indeed, type 1 DM is directly caused by an autoimmune response against pancreatic beta-cells [1,2,3], while chronic subclinical inflammation evidenced in type 2 DM is usually attributed to the proinflammatory activity of adipose tissue [4,5,6,7]. Evidence exists, however, that the level of cytokines in diabetes patients remains high even after weight loss [8]. This fact outlines the important, yet not the exclusive, role that excessive adipose tissue plays in the inflammatory process in type 2 DM. For example, hyperglycemia itself can induce the expression of proinflammatory molecules by β-cells and lead to the activation of fibroblast growth factors and inflammatory markers [9,10]. There are enough data confirming the contribution of low-intensity systemic inflammation to the development and progression of the atherosclerotic disease in individuals with or without impaired carbohydrate metabolism [11,12,13]. Therefore, insulin resistance, dysglycemia, atherosclerosis and chronic inflammation can be considered as links of the same pathogenetic process. It is worth mentioning that the existing studies were mainly focused on the state of lipid metabolism and inflammatory status, as well as their mutual influence, in separate cohorts of patients with either type 1 or type 2 DM. That did not allow for the comparative assessment of the contribution of diabetes-specific metabolic disorders to the development of both systemic inflammation and the disorders of lipid metabolism and ferrokinetics. 

The role of chronic low-grade inflammation in the development of anemic syndrome has been acknowledged. The process involves the promotion of myelopoiesis at the expense of erythropoiesis induced by the cytokines, suppression of erythroid-committed precursor proliferation and macrophage activation for erythrophagocytosis by tumor necrosis factor α (TNF-α) and decrease in iron delivery to plasma from macrophages, which is governed by interleukin 6 (IL-6) through its effects on hepcidin production [14]. At present, studying the features of iron metabolism in individuals with impaired carbohydrate metabolism seems to be rather relevant [15,16,17]. However, quite a few studies to date have investigated the relationship between lipid metabolism and other metabolic parameters, inflammatory status and ferrokinetics in patients with type 1 and type 2 DM in comparative aspects. Therefore, the aim of the present study was to establish the relationship between inflammatory markers, ferrokinetics parameters and lipid metabolism in patients with type 1 and 2 DM.

## 2. Materials and Methods

### 2.1. Study Design

Study design—an observational single-center, one-stage, cross-sectional controlled study. Patients with type 1 and type 2 DM were included in the study during planned hospitalization in the endocrinology clinics of Siberian State Medical University after evaluating the inclusion and exclusion criteria. Diabetes mellitus was diagnosed anamnestically. Patients were stratified into main groups 1 and 2 according to the type of DM. After inclusion in the study, patients underwent a block of all laboratory tests indicated below in the text. The control group included healthy volunteers. Before inclusion in the study, in order to exclude disorders of carbohydrate metabolism, a standard glucose tolerance test with 75 g of glucose was performed for healthy controls. All healthy volunteers of the control group underwent the same laboratory tests as patients with type 1 and type 2 DM.

### 2.2. Inclusion and Exclusion Criteria

*Inclusion criteria for the diabetes groups*. Patients with an established diagnosis of type 1 or type 2 DM and a disease history of 1 to 30 years, aged 18 to 70 years, with glycated hemoglobin level between 6.5% and 10.5% and estimated glomerular filtration rate (eGFR) > 15 mL/min/1.73 m^2^ as assessed by the CKD-EPI creatinine equation (chronic kidney disease stages 1–4), were included in the study.

*Inclusion criteria for the control group*. Patients aged 18 to 70 years, with body mass index (BMI) from 18.5 to 29.9 kg/m^2^. and the absence of carbohydrate metabolism disorders as assessed by glycated hemoglobin concentration and 75 g oral glucose tolerance test, were included in the study.

*Exclusion criteria:* infectious diseases in the acute stage, specific infectious diseases such as: HIV/AIDS; viral hepatitis with any degree of activity; liver cirrhosis of viral or autoimmune etiology; tuberculosis; malignancy; chronic obstructive pulmonary disease or bronchial asthma; active smoking at the time of inclusion in the study; blood transfusion within 1 month prior to the inclusion in the study or at the moment; iron supplements intake; pre or postoperative period; acute renal, hepatic or heart failure; eGFR below 15 mL/min/1.73 m^2^; presence of proteinuria; decompensation of DM manifest in the form of ketoacidosis/hyperosmolar hyperglycemic state; refusal of the patient to participate in the study, refusal to sign the informed consent form.

### 2.3. Methods

The study was conducted on the basis of the endocrinology clinics of Siberian State Medical University, Tomsk. A total of 146 people who underwent planned hospitalizations were enrolled over the span of two years (2017–2019), 48 of which had a diagnosis of type 1 DM (group 1), while 81 had type 2 DM (group 2). The control group consisted of 17 healthy volunteers.

Ten milliliter samples of venous plasma and serum were collected from the cubital vein in the morning after a fasting period using vacutainer tubes. All patients underwent comprehensive anthropometric evaluation. To assess the state of carbohydrate metabolism and its level of compensation, evaluation of glycated hemoglobin concentration was performed using a D10 analyzer (BIO-RAD, Hercules, CA, USA). Serum creatinine concentration was evaluated, with the subsequent calculation of eGFR using the CKD-EPI equation. Quantitative assessment of microalbuminuria (MA) (mg/L) was performed using an Abbott Architect c4000 analyzer (USA). Main hematological parameters (red blood cell count (RBC), reticulocyte count, hemoglobin concentration and hematocrit level) were evaluated using an XN1000 analyzer (Sysmex, Kobe, Japan). Iron metabolism indices (serum iron (μmol/L), transferrin (mg/dL) and ferritin (ng/mL) concentrations) were assessed using an ARCHITECT i2000SR analyzer (Abbott, Abbott Park, IL, USA). Lipid profile values (total cholesterol (TC), high-density lipoprotein cholesterol (HDL-C), low-density lipoprotein cholesterol (LDL-C), very low-density-lipoprotein cholesterol (VLDL-C), and triglyceride (TG) concentrations (mmol/L)) were estimated using an ARCHITECT i2000SR analyzer (Abbott, USA). Atherogenic coefficient was calculated according to the formula TC-HDL cholesterol/HDL cholesterol (TC-HDL-c)/HDL-c). Among the evaluated inflammatory markers were erythrocyte sedimentation rate (ESR) as assessed using an XN1000 hematology analyzer (Sysmex, Japan), high-sensitive C-reactive protein (CRP) (ng/mL), and TNF-α (pg/mL), both assessed by enzyme-linked immunosorbent assay (ELISA) (Vector Best, Novosibirsk, Russia). ESR Hyperlipidemias were classified according to the Fredrickson classification (1967) [18].

### 2.4. Research Ethics 

All subjects gave their informed consent for inclusion before they participated in the study. The study was conducted in accordance with the Declaration of Helsinki, and the protocol was approved by the Ethics Committee of Siberian State Medical University (protocol No. 5596, 06.11.2017)

### 2.5. Statistical Analysis

A sample calculator was used in order to establish the required sample size. The minimum required size of a representative sample for a confidence interval equaling five was estimated to be 70 people. We also used a consistent strategy for calculating the sample size, taking into account the coefficient of variation (= standard deviation from the arithmetic mean in %). According to these calculations, the minimum required sample size for the main groups (patients with DM) was 61 people. Statistical analysis was performed using the SPSS Statistics ver. 23 software package (IBM Corp., Chicago, IL, USA). The Kolmogorov-Smirnov test was used to assess data distribution with the level of significance set at *p* < 0.05. Normally distributed parameters included glycated hemoglobin concentration, eGFR, transferrin, hematocrit and RBC. The remaining parameters, namely: age, duration of diabetes, body mass index (BMI), MA, serum creatinine, CRP, TNF-α, ESR, iron, ferritin, hemoglobin, reticulocyte count, leukocyte count, aspartate aminotransferase (AST), alanine aminotransferase (ALT), TC, HDL, LDL, VLDL and TG, did not obey the normal distribution law. For the sake of comprehensive and unified data presentation, all results were expressed as median and interquartile range (Me, Q0.25–Q0.75). A comparative analysis between two independent groups was performed using the Mann–Whitney criterion with the Bonferroni correction for multiple comparisons, the significance threshold being set at *p* < 0.017, alpha value = 0.05. Student’s *t*-test was used for normally distributed data. Correlations were evaluated using the nonparametric Spearman rank correlation with the significance level was set at *p* < 0.05. Categorical variables were presented as numbers and percentages. Spearman’s chi-square test was applied with a 5% significance level to test for differences between them. 

## 3. Results

The study included 129 patients with either type 1 or type 2 DM, and 17 healthy volunteers with normal BMI and no evidence of carbohydrate metabolism disorders.

Among people with DM, 43 were men (33.3%) and 86 (66.7%) women. Within the main groups (groups 1 and 2), the ratio of men and women was comparable: in the group of patients with type 1 DM there were 19 (39.6%) men and 29 (60.4%) women, while the group of patients with type 2 DM comprised 24 (29.6%) men and 57 (70.4%) women (χ^2^ = 1.276; *p* = 0.259). The control group, just like the main groups, had fewer men than women (4 (23.5%) and 13 (76.5%), respectively). Table 1 shows the clinical characteristics of the study groups.

As shown in Table 1, groups 1 and 2 were comparable in terms of DM duration, glycated hemoglobin concentration and MA. At the same time, patients with type 2 DM had significantly higher BMI compared to patients with type 1 DM (*p* ˂ 0.0001) and control subjects (*p* ˂ 0.0001). eGFR in patients with type 2 DM was lower than in patients with type 1 DM (*p* = 0.006) and healthy volunteers (*p* ˂ 0.0001). Glycated hemoglobin concentration in the control group was significantly lower compared to patients with type 1 and type 2 DM (*p* < 0.0001 in both cases).

Among patients with DM, 94 people (72.8%) were suffering from hypertension at the time of inclusion in the study. Of these, 13 people (13.8%) did not receive antihypertensive treatment, while the remaining 81 patients (86.2%) were taking 1 to 4 hypotensive agents. People from the control group did not suffer from arterial hypertension.

At the time of inclusion, of all patients with DM, 106 (82.2%) people had no history of acute cardiovascular events (myocardial infarction/stroke), 17 (13.2%) patients had a history of myocardial infarction, five (3.8%) people had the anamnesis of stroke, and one patient (0.8%) suffered from both stroke and myocardial infarction. Cardiovascular events were more prevalent in patients with type 2 DM compared to individuals with type 1 DM (χ^2^ = 8.049; *p* = 0.045).

All patients with type 1 DM received basal-bolus insulin therapy. Among patients with type 2 DM, 22 people (26.2%) took oral hypoglycemic agents, 19 people (22.6%) received various modes of insulin therapy, while in 43 patients (51.2%) insulin was used in combination with oral antidiabetic agents. 

As a result of a comparative analysis of inflammatory status, it has been shown that TNF-α level was significantly higher in patients with type 1 DM when compared to both patients with type 2 DM (*p* < 0.0001) and individuals from the control groups (*p* = 0.004). On the contrary, patients with type 2 DM demonstrated significantly higher CRP concentrations than patients with type 1 DM (*p* ˂ 0.0001). Assessment of CRP in the control group was not performed due to technical reasons. It is worth noting that in type 2 DM patients, the ESR was also significantly higher than in their counterparts with type 1 diabetes mellitus (*p* ˂ 0.0001) and in people from the control group (*p* ˂ 0.0001).

Significant differences between groups were found in the concentration of ferritin, level being significantly higher in patients with type 2 DM compared to patients with type 1 DM (*p* = 0.013). There were no significant differences in other parameters of iron metabolism between the groups. The results of a comparative assessment of inflammatory status and ferrokinetics are shown in Table 2.

Significant differences in certain indices of lipid profile were revealed depending on the presence and type of DM. In particular, patients with type 2 DM had significantly higher levels of VLDL-C (*p* < 0.0001), TG (*p* < 0.0001) and atherogenic coefficient values (*p* < 0.0001, as well as lower concentrations of HDL-C (*p* < 0.0001), when compared with type 1 DM patients (Table 3). At the same time, there were no significant differences in lipid profiles of patients with type 1 DM and healthy volunteers of the control group (Table 3). Comparative characteristics of lipid profile in three groups are presented in Table 3.

Taking into account the statistically significant differences in age of patients with type 1 and type 2 DM, as well as the correlations between age and lipid metabolism obtained during further research, it can be assumed that the differences in cholesterol and its components are caused not only by the type of diabetes, but the influence of age as well. 

Among all persons included in the study (*n* = 146), hyperlipidemia was detected in 80.1% (*n* = 117). It is worth mentioning, however, that hyperlipidemia was not detected in any of the patients from the control group. Hyperlipidemia was present in 83.3% of patients with type 1 DM, and in 95.1% of type 2 DM cases (Table 4).

Among patients with hyperlipidemia (*n* = 117), only 27.4% (*n* = 32) received lipid-lowering therapy at the time of inclusion in the study. The only group of pharmacological agents received by the patients in our study were statins. Frequency of statin intake varied between groups, from 12.5% (*n* = 5) in patients with type 1 DM, to 35.1% (*n* = 27) in individuals with type 2 DM.

In patients with hyperlipidemia not receiving lipid-lowering drugs at the time of inclusion in the study (*n* = 91), Fredrickson’s classification was employed to assess the type of lipid metabolism disorder. Analysis revealed the predominance of highly atherogenic type IIb hyperlipidemia in patients with type 2 DM. On the other hand, in patients with type 1 DM, less atherogenic phenotype of hyperlipidemia (type IIa) was more common (χ^2^ = 34.051; *p* < 0.0001).

Spearman’s coefficient was calculated to assess correlations between lipid profile, parameters of metabolic control, markers of chronic inflammation, and iron metabolism indices in an overall sample of patients with DM (*n* = 129), as well as in individual groups of type 1 (*n* = 48) and type 2 (*n* = 81) DM, and in healthy controls (*n* = 17). The relevant data are presented in Table 5, Table 6, Table 7 and Table 8.

Statistical analysis revealed a negative correlation between HDL-C concentration and microalbuminuria level in the overall sample of DM patients, which may reflect the mechanism of development and progression of endothelial dysfunction and, as a consequence, microvascular complications, as well as the role hyperlipidemia plays in this process (Table 5). In addition, regardless of DM type, there was a positive correlation between triglyceridemia and serum ferritin level. The mechanism of this relationship may be partly explained by the effects of functional activity of adipose tissue and an increase in the production of free fatty acids. Subsequently, this would lead to the development of nonalcoholic steatohepatitis, in which the inflammatory mesenchymal reaction would contribute to hyperferritinemia.

Type 1 DM-specific correlations were also established (Table 6). For instance, TC levels and all of its fractions, with the exception of HDL-C, as well as the atherogenic coefficient, demonstrated positive correlations with serum creatinine and negative ones with eGFR. In addition, VLDL-C levels were positively correlated with microalbuminuria. These correlations reflect the role of hyperlipidemia in the progression of endothelial dysfunction and the development of microvascular complications, in particular, diabetic nephropathy. Positive relationships were also established between markers of chronic inflammation and the lipid profile parameters TC, VLDL-C and TG levels positively correlated with ESR, while CRP concentration positively correlated with TG level and atherogenic coefficient. The relationships between leucocyte count and VLDL-C and atherogenic coefficient were also revealed. In addition, positive correlations were observed between concentration of serum ferritin and TC, LDL-C and TG levels. As is well known, ferritin not only reflects total amount of iron stored in the body, but also acts as one of the acute phase proteins, with concentration increases in inflammation. Thus, it may be noted that in patients with type 1 DM, hyperlipidemia with an increase in atherogenic fractions of cholesterol is associated with chronic subclinical inflammation.

As for the group of type 2 DM patients, there was a positive relationship between BMI and VLDL-C and triglyceride levels. On top of that, negative correlation was revealed between BMI and HDL-C concentration, which reflects the role of adipose tissue in the development of hyperlipidemia. In addition, negative correlations between reticulocytes content and levels of TC and LDL-C were observed. At the same time, associations between inflammatory markers and indices of lipid profile were not characteristic for this group of patients (Table 7).

Correlations identified in the control group can be found in Table 8. 

Taking into account the statistically significant age differences in patients with type 1 and type 2 DM, correlations between age and the indicators of the lipid profile were studied. The result of studying these correlations was rather expected—in patients with DM there were positive correlations between age and the concentrations of atherogenic fractions of cholesterol, and a negative correlation between age and HDL-C concentration (only for type 1 DM). In the healthy control group, a positive correlation between age and TC remained. Moreover, in the overall sample of patients with DM, as well as in the group of people with type 2 DM, there was a weak correlation (*r* = 0.175 and *r* = 0.278, respectively *p* < 0.05) between age and ESR, which may confirm the potential effect of age on the severity of chronic subclinical inflammation. No other statistically significant correlations between age and iron metabolism and inflammation parameters were detected.

Thus, distinct correlations between the parameters of metabolic control, iron metabolism and chronic inflammation were obtained in both overall sample of people with DM and in patients suffering from specific types of DM (either type 1 or type 2).

## 4. Discussion

As a result of the study, it was possible to establish general (universal) relationships between the parameters of lipid profile and metabolic control in DM, regardless of its type, namely:

- a negative relationship between HDL concentration and the level of microalbuminuria, reflecting the primary role of HDL deficiency, rather than the increase in atherogenic lipid fractions, in the development and progression of endothelial dysfunction, ultimately leading to the development and exacerbation of diabetic nephropathy. The role of diabetic nephropathy in lowering plasma level of HDL-C was been established. Both hypotheses take into account the existing literature data;
- a positive correlation between triglyceridemia and serum ferritin concentration. This relationship can be explained by the effects of functional activity of adipose tissue and an increase in the production of free fatty acids leading to the development of nonalcoholic steatohepatitis with increase in the inflammatory mesenchymal reaction of the liver.

It is worth mentioning that a positive correlation between TG levels and serum ferritin content was evident not only in patients with type 1 and type 2 DM, but also in people from the healthy control group. The aforementioned control group subjects did not have hyperlipidemia or impaired carbohydrate metabolism. Therefore, the presence of this relationship in this subset of people characterizes it as strictly determined, resulting from the strong interdependence between the amount of adipose tissue, the level of free fatty acids and subsequent development of chronic liver inflammation and hyperferritinemia.

In patients with type 1 DM, atherogenic hyperlipidemia was associated with impaired renal function as assessed by decreased GFR, increased microalbuminuria and serum creatinine concentration, and chronic subclinical inflammation (evidenced by the increased ferritin, ESR and CRP). Revealed correlations are in agreement with the literature data and reflect the role of hyperlipidemia and chronic inflammation in progression of endothelial dysfunction and the development of microvascular complications such as diabetic nephropathy.

In the group of patients with type 2 DM, in addition to the universal relationships already noted previously, positive associations were established between an increase in atherogenic lipid fractions and BMI, which confirms the classical theory of the role of adipose tissue in the development of hyperlipidemia.

The results obtained in our study are in agreement with the existing literature data from both single-center comparative studies and multicenter cohort observational studies. Thus, a study conducted by Palvasha Waheed et al. showed the presence of a significant positive correlation between the inflammatory markers, hs-CRP and ferritin, and the parameters of dyslipidemia—TC, LDL-C and TG (*p* < 0.001, *r* = 0.72) except for HDL-C, which had an insignificant negative correlation with the inflammatory markers (*p* > 0.05 *r* = −0.10) [19]. According to the DCCT/EDIC study, lower LDL-C and TG levels were associated with reduced risk for progression from moderate albuminuria to severe albuminuria or end-stage renal disease [20]. A 2015 literature review provides a series of studies searching for new risk factors for developing diabetic nephropathy [21]. Among these factors, higher levels of proinflammatory cytokines and chemokines (interleukin 6, interleukin 18, and monocyte chemoattractant protein−1, hsCRP) are noted [22,23].

A large cross-sectional study (China Health and Nutrition Survey 2009) found that elevated serum ferritin levels were associated with the prevalence of hyperlipidemia among Chinese adults. There was a significant positive association between serum ferritin levels and lipid parameters independent of diabetes and insulin resistance in both genders. This study also showed that subjects with hyperlipidemia and diabetes had higher serum ferritin levels than subjects without hyperlipidemia and diabetes [24]. These results are in agreement with the results of correlation analysis conducted as part of our study.

It is well established that type 1 and type 2 DM are characterized by distinct lipid metabolism disorders manifested in the form of specific shifts in lipid profile. For instance, type 2 DM is characterized by a high TG level, low HDL-C concentration and denser LDL particles. Hypertriglyceridemia in type 2 DM develops as a result of insulin resistance and abdominal obesity. These changes serve as the background for the increase in the serum concentration of free fatty acids due to their augmented release from adipose tissue and reduced muscle consumption. As a response to these changes, liver increases production of VLDL and saturated TG, while the lipoprotein lipase-mediated hydrolysis of VLDL decreases. This ultimately leads to an increase in levels of TG-rich VLDL. The decrease in HDL-C concentration in type 2 DM is secondary, being attributed to the increased transfer of cholesterol esters from HDL to VLDL in exchange for TG. TG-saturated HDL are then rapidly destroyed by hepatic lipase. Moreover, in type 2 DM, the ability of HDL to inhibit LDL oxidation is disrupted, while the number of functionally defective HDL increases. Among the most important nonlipid proatherogenic factors in type 2 DM are oxidative stress due to overproduction of reactive oxygen species, accumulation of advanced glycation end products within the walls of blood vessels, increased endothelin production under the effect of hyperinsulinemia and apoptosis of smooth muscle cells of vascular walls. Combinations of these factors promote diffuse generalized endothelial dysfunction [25,26,27]. 

In patients with type 1 DM, lipid metabolism disorders are less common and often less pronounced. In the setting of adequate glycemic control, levels of TG and LDL-C are reduced in this category of patients. In addition, insulin therapy can increase the level of HDL-C, which is caused by stimulation of lipoprotein lipase activity in adipose tissue and skeletal muscles, resulting in the intense metabolism of VLDL. Furthermore, several studies have consistently shown that the severity of dyslipidemia increases after the development of diabetic nephropathy, manifesting in the form of elevated TG level and decrease in HDL-C concentration [28].

The significant differences obtained in the course of our study between the concentration of the lipid profile parameters in patients with type 1 and type 2 DM are consistent with the above literature data, though could also be influenced by age in our patient sample.

Glomerular mesangial cells, just like smooth muscle cells, express cell-surface LDL receptors. Under conditions of hyperlipidemia, these cells are capable of capturing and accumulating LDL and their oxidized forms. The presence of oxidized LDL promotes infiltration of mesangium by mononuclear cells and macrophages, which produce cytokines and growth factors. Oxidized LDL, growth factors and cytokines cause an increase in the synthesis of mesangial matrix and basement membrane components, thus advancing the development of glomerular sclerosis. In addition, lipoproteins can stimulate the activation of the transforming growth factor β (TRF-β) signaling pathway, which, in turn, promotes the production of reactive oxygen species that cause glomerular damage. In addition to the TRF-β signaling pathway, LDL have demonstrated the ability to activate monocytes and destroy cellular glycocalyx, causing increased glomerular permeability [29,30].

It should be noted that the development of diabetic nephropathy not only exacerbates atherogenic hyperlipidemia, but also accelerates the progression of endothelial dysfunction. As kidney function decreases, the synthesis of Apolipoprotein AI (ApoA-I) in the liver, which is the main HDL component, drops accordingly, leading to a decrease in the plasma level of HDL-C. Apo A-I is also an important activator of lecithin-cholesterol acyltransferase, an enzyme crucial for the conversion of HDL-3 to cholesterol-rich HDL-2. Inflammation of the vascular wall can subsequently cause structural and functional disorders of HDL. Thus, the most important antiatherogenic functions of HDL are disrupted, which in turn leads to a predisposition of vessels to oxidative stress [31]. According to published data, normalization of HDL levels can lead to the reduction in the risk of diabetes complications, in particular micro and macroangiopathies.

The suggested presence of shared molecular mechanisms of inflammation and insulin signaling pathways [32], supposedly resulting in insulin resistance, endothelial dysfunction and cardiovascular complications, provides a theoretical basis for the hypothesis of common causal factors for both diabetes and atherosclerosis (the theory of common soil) [33]. The concept that inflammation participates pivotally in the pathogenesis of atherosclerosis and its complications has gained considerable attention but has not yet entered clinical practice.

The literature review published in 2016 notes potential interventions aimed at preventing the progression of diabetic nephropathy, such as the use of pentoxifylline, silymarin, the endothelin-1A antagonist atrasentan, octreotide and statins [34]. In this case the use of statins has the most powerful evidence base.

It is worth mentioning that many studies have noted the effect lipid-lowering therapy exerts on the markers of chronic inflammation in DM patients. For example, in the CARE (Cholesterol And Recurrent Events) study, it was first shown that statin therapy causes a decrease in both LDL-C and CRP levels. Thus, after 5 years of pravastatin treatment, CRP concentration decreased by 35% compared with placebo [35]. According to the results of the PRINCE (pravastatin inflammation/CRP evaluation) study, pravastatin administration resulted in a decrease in CRP level by 15% within 12 weeks after the start of therapy [36]. In the JUPITER (Justification for the Use of Statins in Primary Prevention: An Intervention Trial Evaluating Rosuvastatin) trial rosuvastatin reduced both median LDL-cholesterol by 50% and hsCRP by 37% [37]. At the same time, the ability of fenofibrate to normalize HDL-C levels was demonstrated. Moreover, DAIS (Diabetes Atherosclerosis Intervention Study) [38] and FIELD (Long-term fenofibrate therapy and cardiovascular events in people with type 2 diabetes mellitus) [39] studies showed decreases in the rate of occurrence and progression of microalbuminuria in patients receiving fenofibrate therapy. 

Thus, the traditional use of renin–angiotensin–aldosterone system (RAAS) blockers alone is insufficient for preventing the progression of diabetic kidney disease in a large subset of patients. The results of our study are consistent with literature data, including those obtained in multicenter cohort studies, and suggest that it is necessary to develop an earlier and broader algorithm of action, as opposed to the existing clinical guidelines, for prescribing statins to patients with type 1 DM, with the aim of preventing the development and progression of diabetic nephropathy

## 5. Conclusions

Established relationships between the parameters of lipid profile, inflammatory status and microalbuminuria confirm the mutual influence of hyperlipidemia, chronic inflammation and nephropathy in DM. In addition, obtained results allow us to consider and justify a strategy for early hypolipidemic therapy initiation in patients with DM, including type 1 DM, from the point of view of preventing the development and progression of microvascular complications, diabetic nephropathy in particular.

The limitations of this study include the relatively small number of controls, which is planned to increase in the course of further research. Age differences between patients with type 1 and type 2 DM can also be considered as a limitation of this study. However, the formation of groups comparable in age can lead to the occurrence of statistical differences in the duration of DM or the severity of microvascular complications, including diabetic nephropathy between these groups. 

Future perspectives of the study also include the study of the prospects for earlier intervention by means of lipid-correcting therapy for the course of diabetic nephropathy and the development and progression of anemic syndrome.

## Figures and Tables

**Table 1:** Clinical characteristics of patients in the studied groups.

Variables	Type 1 DM n = 48	Type 2 DM n = 81	Control Group (Healthy Individuals) n = 17
Age, years	34.00 (26.00–52.00) **	60.00 (56.00–65.00) *	40.00 (32.00–58.00)
Duration of the disease, years	9.0 (3.00–17.00)	11.00 (8.00–15.00)	--
BMI, kg/m^2^	23.67 (21.43–26.03) **	33.80 (29.55–38.82) *	25.10 (23.10–27.65)
HbA1c, %	8.80 (6.95–10.30) *	9.10 (7.97–11.03) *	5.20 (4.90–5.85)
eGFR, mL/min/1.73 m^2^	95.00 (71.75–112.75) **	80.50 (63.00–93.00) *	96.50 (93.00–106.00)
MA, mg/L	20.50 (9.25–39.25)	13.55 (8.53–30.00)	--
AST (IU/L)	20.00 (16.60–27.00)	19.40 (15.00–28.00)	20.00 (16.50–22.50)
AST (IU/L)	16.00 (12.00–24.00) **	20.00 (14.00–29.75)	18.00 (11.50–21.00)

*—significant differences when compared to control group (*p* ˂ 0.017); **—significant differences when compared to group 2.

**Table 2:** Inflammatory markers and iron metabolism indices in patients with diabetes mellitus (DM) and in the control group.

Variables	Type 1 DM *n* = 48	Type 2 DM *n* = 81	Control Group (Healthy Individuals) *n* = 17
TNF-α, pg/mL	15.28 (12.41–24.41) *^,^**	8.54 (6.27–11.60)	9.68 (5.68–15.38)
CRP, ng/mL	2.00 (1.05–4.05) **	7.00 (3.00–11.85)	--
ESR, mm/h	14.00 (5.00–21.25) **	18.00 (9.00–27.00) *	7.00 (5.00–9.00)
Leucocyte count, ×10^9^/L	6.55 (5.30–7.83)	7.38 (6.08–8.74)	6.08 (5.25–7.53)
Hemoglobin, g/L	138.50 (122.50–151.00)	141.00 (125.25–151.00)	146.00 (135.00–150.00)
Erythrocyte count, ×10^12^/L	4.69 (4.38–5.09)	4.79 (4.39–5.19)	4.80 (4.49–5.02)
Reticulocytes, %	1.51 (1.12–1.75)	1.76 (1.54–1.91)	1.60 (1.40–1.66)
Hematocrit, %	40.95 (38.40–43.65)	42.05 (38.00–44.55)	42.70 (40.70–44.85)
Iron, μmol/L	12.00 (8.00–17.00)	13.00 (11.00–18.25)	16.00 (11.00–20.50)
Ferritin, ng/mL	44.48 (18.35–148.50) **	96.52 (42.93–189.0)	72.05 (43.23–148.60)
Transferrin, mg/dL	284.00 (250.00–334.00)	293.00 (267.00–321.50)	267.50 (208.75–306.50)

*—significant differences when compared to control group (*p* < 0.017); **—significant differences when compared to group 2.

**Table 3:** Comparative characteristics of the lipid profile in patients with DM and in the control group.

Variables	Type 1 DM *n* = 48	Type 2 DM *n* = 81	Control Group (Healthy Individuals) *n* = 17
TC, mmol/L	4.98 (4.33–5.68)	5.41 (4.58–6.40)	4.90 (4.50–5.35)
HDL-C, mmol/L	1.50 (1.23–1.84) **	1.04 (0.90–1.30) *	1.60 (1.33–1.90)
LDL-C, mmol/L	2.95 (2.55–3.28)	3.25 (2.28–4.00) **	3.00 (2.25–3.24)
VLDL-C, mmol/L	0.50 (0.36–0.68) **	1.00 (0.73–1.31) *	0.41 (0.29–0.70)
TG, mmol/L	1.05 (0.73–1.58) **	2.20 (1.60–2.70) *	0.90 (0.65–1.45)
Atherogenic coefficient	2.38 (1.80–3.83) **	4.00 (2.95–5.11) *	2.30 (1.88–2.85)

*—significant differences when compared to control group (*p* < 0.017); **—significant differences when compared to group 2.

**Table 4:** Frequency of hyperlipidemia in patients with DM and in the control group.

Variables	Total (*n* = 146)	Type 1 DM *n* = 48	Type 2 DM *n* = 81	Control Group (Healthy Individuals) *n* = 17
Hyperlipidemia, %(n)	80.1 (117)	83.3 (40)	95.1 (77)	0.0 (0)
Absence of hyperlipidemia, %(n)	19.8 (29)	16.7 (8)	4.9 (4)	100.0 (17)

**Table 5:** Correlations between lipid profile, inflammatory markers and parameters of iron metabolism in patients with DM (independent of type).

			TC	HDL-C	LDL-C	VLDL-C	TG	Atherogenic Coefficient
	Age	*r*	0.235 *	−0.287 *	0.256 *	ns	ns	0.308 *
**Spearman *r***	BMI	*r*	ns	−0.561 *	ns	0.529 *	0.524 *	0.502 *
	eGFR	*r*	−0.187 *	0.233 *	ns	−0.362 *	−0.385 *	−0.256 *
	ESR	*r*	0.200 *	ns	0.200 *	0.261 *	0.271 *	0.233 *
	TNF-α	*r*	ns	0.298 *	ns	−0.343 *	ns	−0.325 *
	CRP	*r*	ns	ns	ns	ns	0,276 *	ns
	Leucocytes	*r*	ns	−0.324 *	ns	0.322 *	0.238 *	0.253 *
	Ferritin	*r*	ns	−0.325 *	ns	0.365 *	0.415 *	0.402 *
	Transferrin	*r*	ns	0.362 *	ns	ns	ns	ns
	Reticulocytes	*r*	−0.346 *	−0.325 *	−0.504 *	ns	ns	ns
	ALT	*r*	ns	−0.200 *	ns	0.321 *	0.315 *	0.237 *

*r*—Spearman’s rank correlation coefficient; ns – nonsignificant differences; *—*p* < 0.05.

**Table 6:** Correlations between lipid profile, inflammatory markers and parameters of iron metabolism in patients with type 1 DM.

			TC	HDL-C	LDL-C	VLDL-C	TG	Atherogenic Coefficient
	Age	*r*	0.436 *	ns	0.407 *	ns	ns	ns
**Spearman *r***	eGFR	*r*	−0.618 *	ns	−0.480 *	−0.490 *	−0.533 *	−0.459 *
	Creatinine	*r*	0.442 *	ns	0.417 *	0.387 *	0.436 *	0.550 *
	MA	*r*	ns	−0.293 *	ns	0.339 *	ns	ns
	ESR	*r*	0.371 *	ns	ns	0.642 *	0.546 *	ns
	CRP	*r*	ns	ns	ns	ns	0.567 *	0.592 *
	Leucocytes	*r*	ns	−0.331 *	ns	0.406 *	ns	0.391 *
	Ferritin	*r*	0.384 *	ns	0.361 *	ns	0.346 *	ns
	Transferrin	*r*	ns	0.490 *	ns	ns	ns	ns
	ALT	*r*	ns	ns	ns	ns	0.363 *	ns

*r*—Spearman’s rank correlation coefficient; ns—nonsignificant differences; *—*p* < 0.05.

**Table 7:** Correlations between lipid profile, inflammatory markers and parameters of iron metabolism in patients with type 2 DM.

			TC	HDL-C	LDL-C	VLDL-C	TG	Atherogenic Coefficient
	Age	*r*	ns	ns	ns	0.299 *	0.233 *	ns
**Spearman *r***	BMI	*r*	ns	−0.326 *	ns	0.255 *	0.230 *	ns
	MA	*r*	ns	−0.272 *	ns	ns	ns	ns
	TNF-α	*r*	ns	−0.440 *	ns	ns	ns	ns
	Ferritin	*r*	ns	−0.328 *	ns	ns	0.244 *	0.328 *
	Reticulocytes	*r*	−0.505 *	ns	−0.496 *	ns	ns	ns

*r*—Spearman’s rank correlation coefficient; ns—nonsignificant differences; *—*p* < 0.05.

**Table 8:** Correlations between lipid profile, inflammatory markers and parameters of iron metabolism in healthy controls.

			TC	HDL-C	LDL-C	VLDL-C	TG	Atherogenic Coefficient
	Age	*r*	0.519 *	ns	ns	ns	ns	ns
**Spearman *r***	BMI	*r*	ns	ns	ns	ns	ns	0.591 *
	Leucocytes	*r*	ns	−0.617 *	ns	ns	ns	ns
	Ferritin	*r*	ns	ns	ns	0.661 *	0.674 *	0.583 *
	Transferrin	*r*	ns	0.572 *	ns	ns	ns	ns

*r*—Spearman’s rank correlation coefficient; ns—nonsignificant differences; *—*p* < 0.05.
